# Supplementary material for: Asymptomatic COVID‐19: disease tolerance with efficient anti‐viral immunity against SARS‐CoV‐2
Source: EMBO Mol Med. 2021 May 27;13(6):e14045. doi: 10.15252/emmm.202114045 (PMC8185544; doi:10.15252/emmm.202114045)
Supplement: Supplementary file 1 — Appendix [file EMMM-13-e14045-s001.pdf]

EMM-2021-14045 Appendix

Appendix Figure S1 – Mass cytometry analyses of PBMCs from COVID-19 patients by Cytometry by time-of-flight (CyTOF).

Appendix Figure S2 – Gating strategy for isolation of neutrophils.

Appendix Figure S3 – Gating strategy for flow cytometry panel A.

Appendix Figure S4 – Gating strategy for flow cytometry panel B.

Appendix Figure S5 – Mass cytometry analyses of monocytes from COVID-19 patients by Cytometry by time-of-flight (CyTOF).

Appendix Table S1 – Flow cytometry antibodies per panels

Appendix Table S2 – Summary of actual P values for Main, Expanded View and Appendix figures.

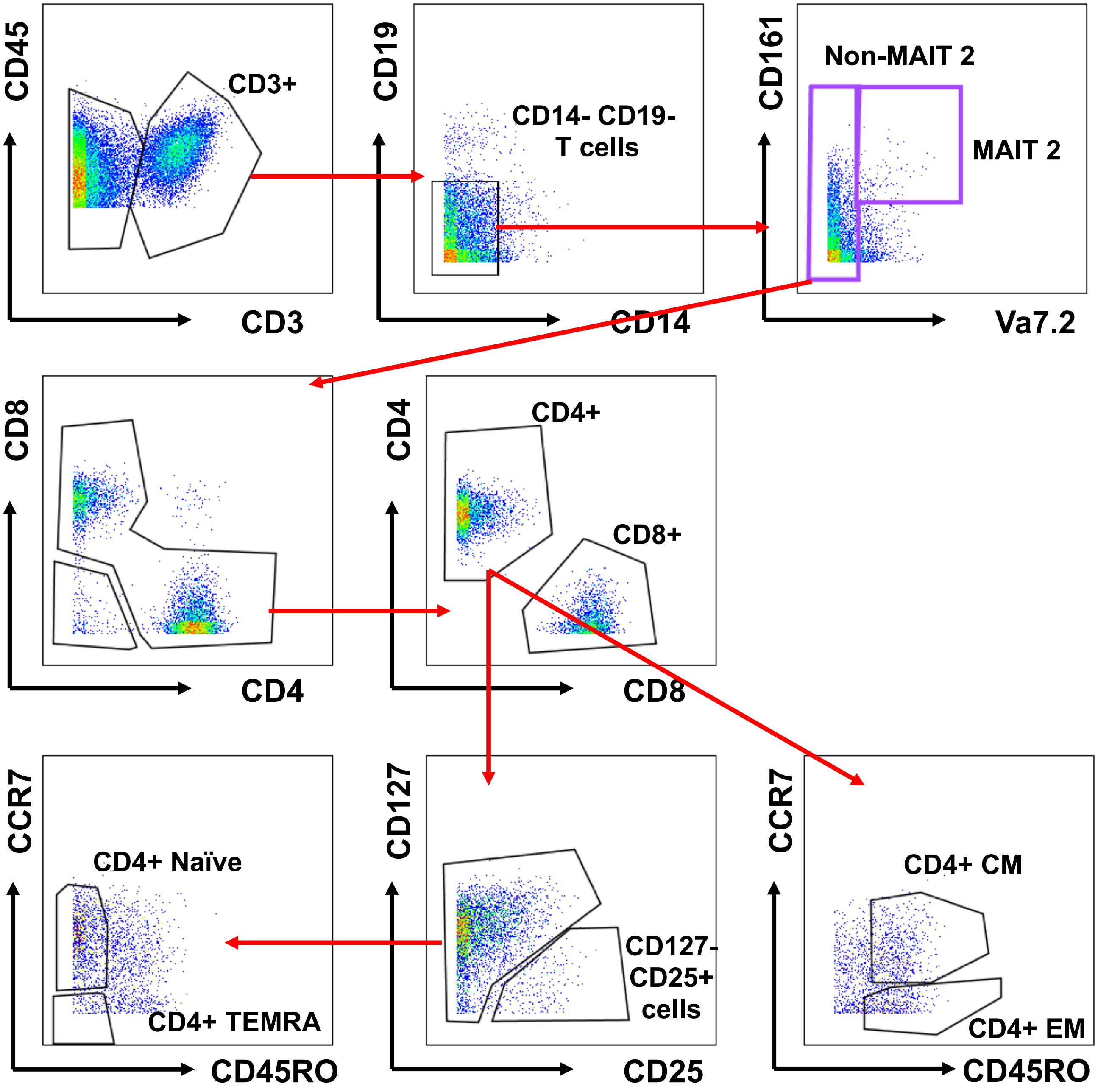

Appendix Figure S1, Chan et al., 2021

Appendix Figure S1 – Mass cytometry analyses of PBMCs from COVID-19 patients by Cytometry by time-of-flight (CyTOF). Representative gating strategy for the characterization of CD4+ T cells in isolated PBMCs of COVID-19 patients. Representative gating strategy was performed on a symptomatic patient.

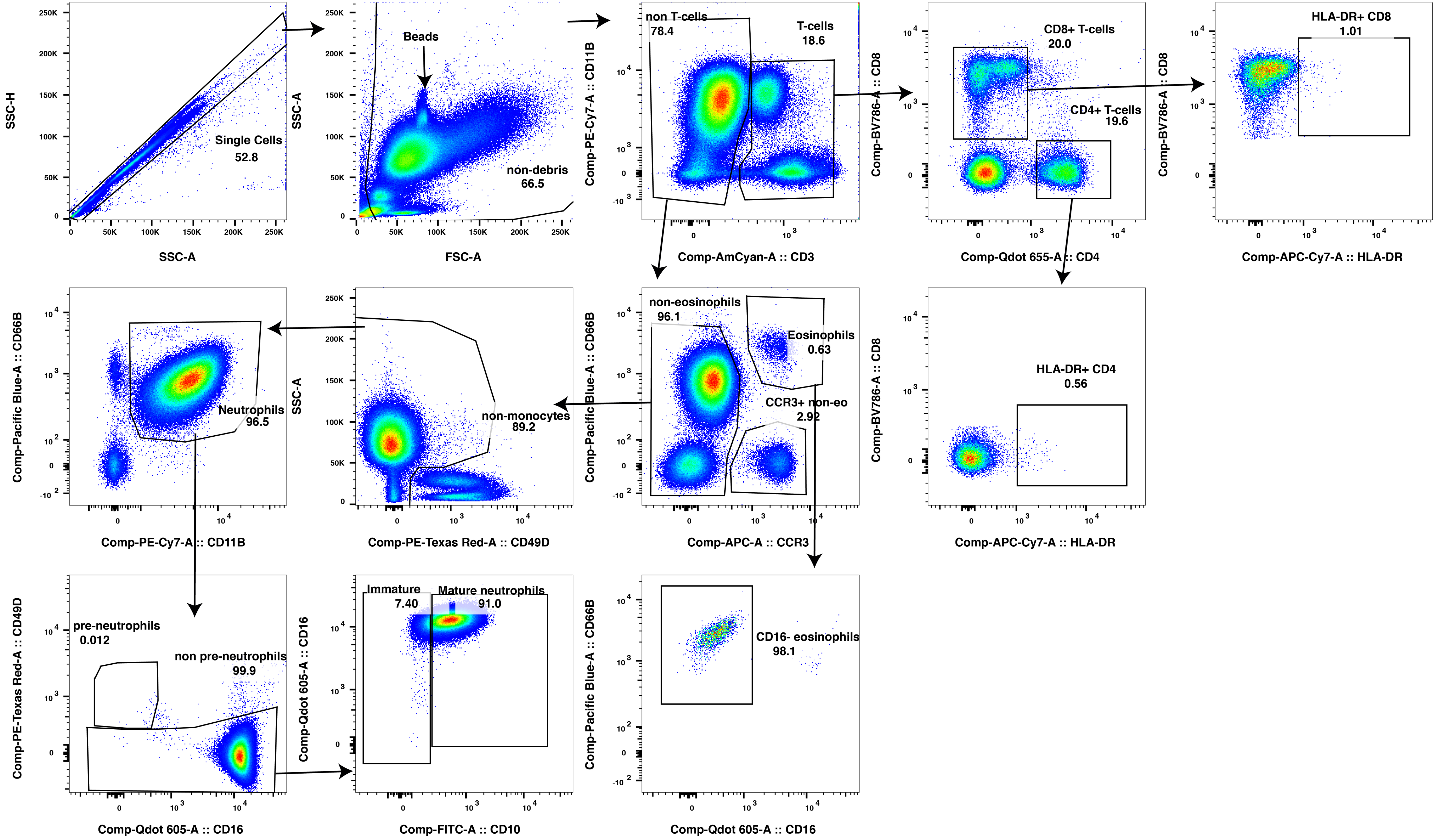

Appendix Figure S2, Chan et al., 2021

Appendix Figure S2 – Gating strategy for isolation of neutrophils.

Immune cells in 100μL of fresh whole blood from acute symptomatic and asymptomatic patients were stained with the antibodies of panel C described in Appendix Table S1. Gating was performed after compensation adjustment in FlowJo as presented in this figure.

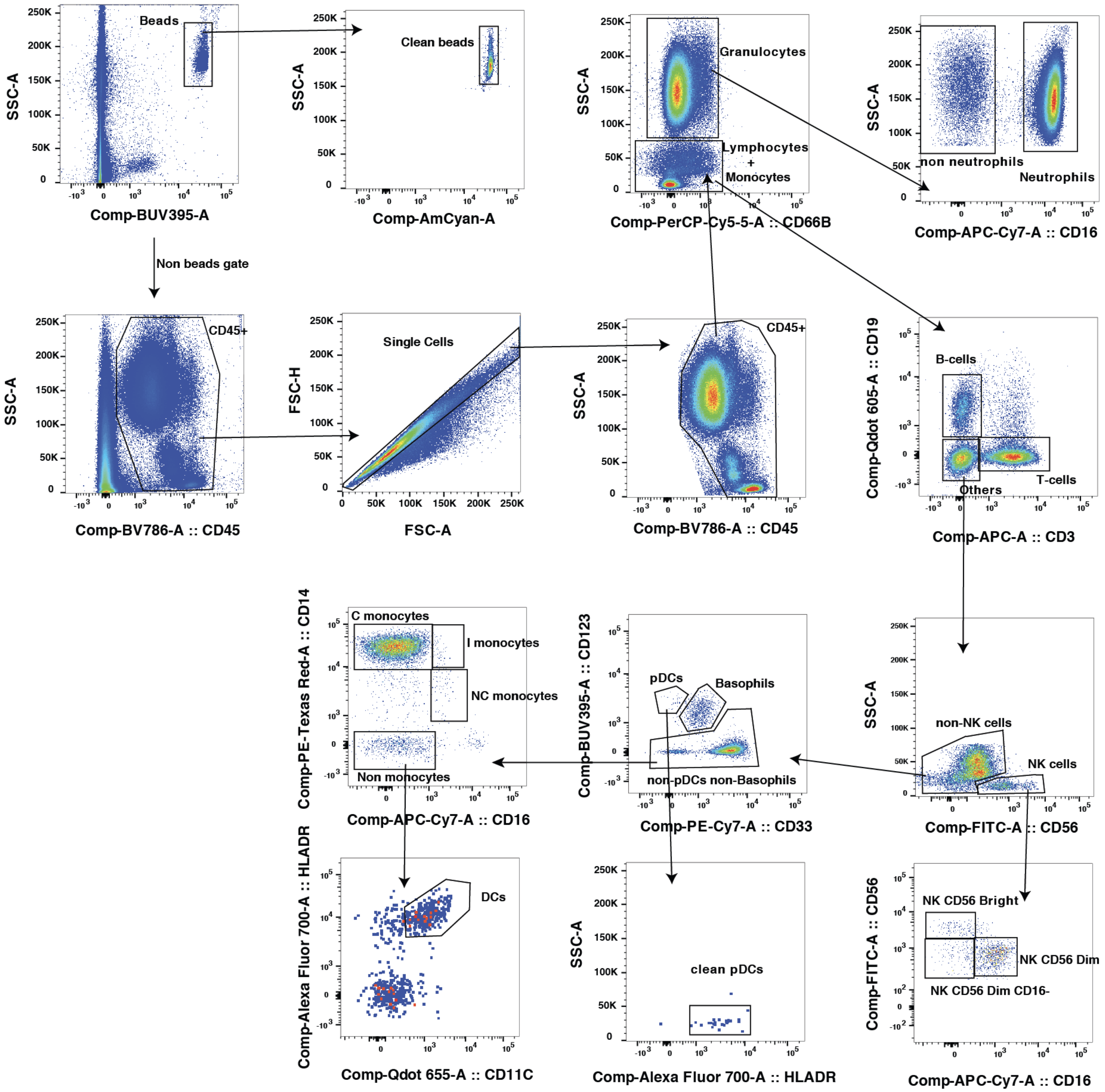

Appendix Figure S3, Chan et al., 2021

Appendix Figure S3 – Gating strategy for flow cytometry panel A.

Immune cells in 100μL of blood was stained with the antibodies of panel A described in Appendix Table S1. Gating was performed after compensation adjustment in FlowJo as presented in this figure.

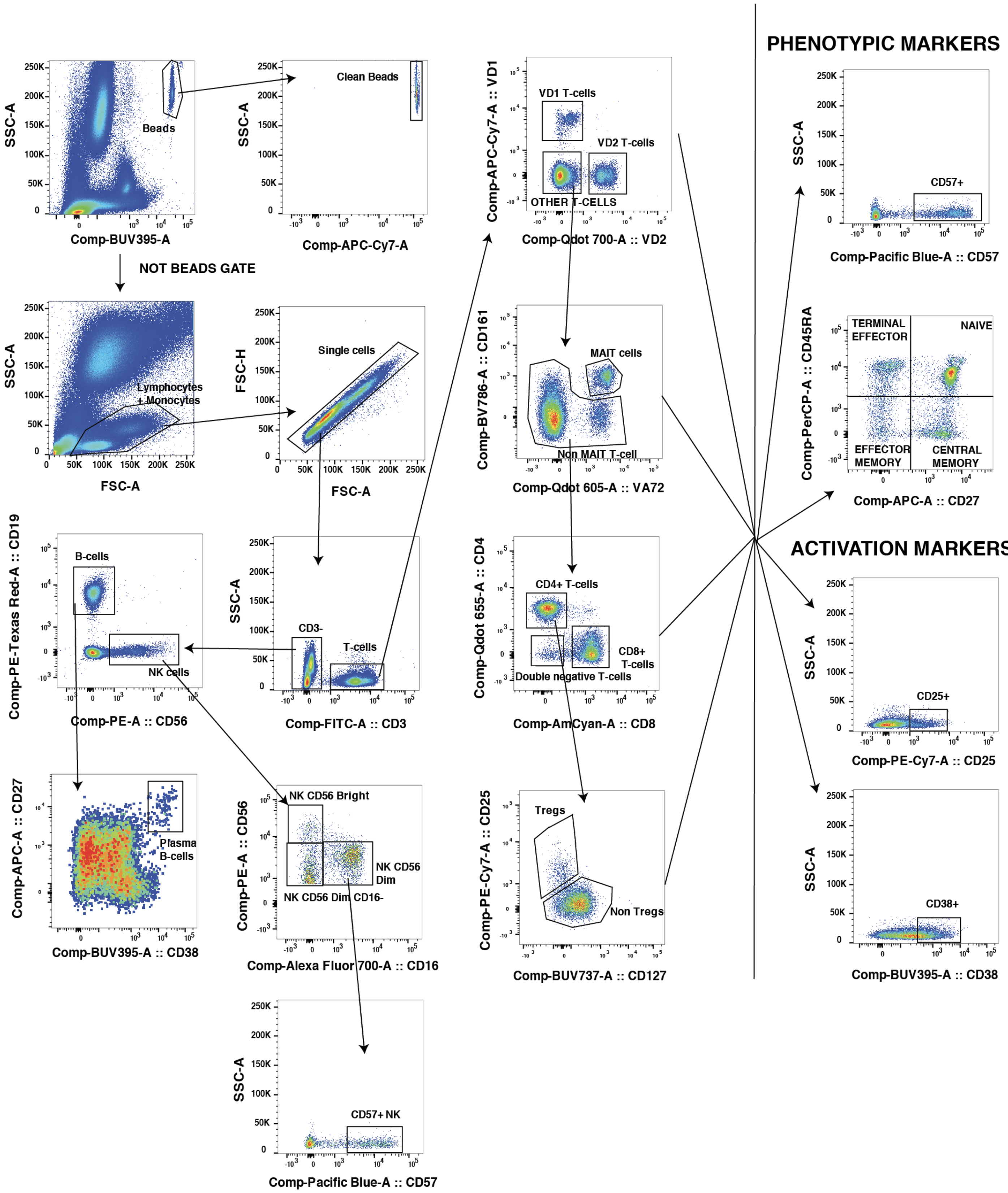

Appendix Figure S4, Chan et al., 2021

Appendix Figure S4 – Gating strategy for flow cytometry panel B.
Immune cells in 100μL of blood was stained with the antibodies of panel B described in Appendix Table S1. Gating was performed after compensation adjustment in FlowJo as presented in this figure.

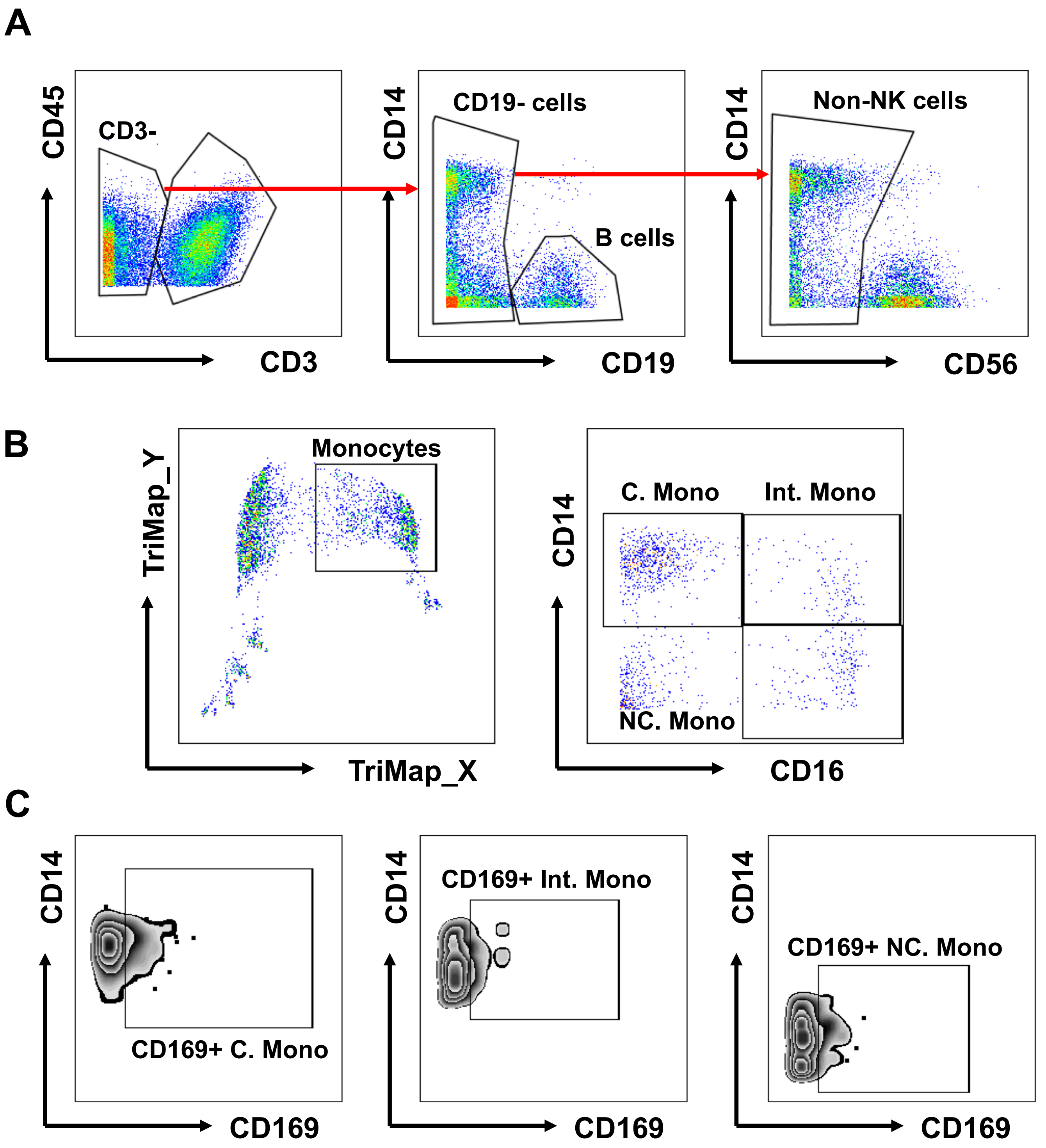

Appendix Figure S5, Chan et al., 2021

Appendix Figure S5 – Mass cytometry analyses of monocytes from COVID-19 patients by Cytometry by time-of-flight (CyTOF).

A. Representative gating strategy for the characterization of monocytes in patient PBMCs. Representative plots are performed with a symptomatic patient. Non-NK cells were isolated from non-B and T cells.

B. Dimensionality reduction method TriMap was performed on the non-NK cells to isolate the monocytes from the low density neutrophils (LDN). Monocytes were then characterized into classical monocytes (C. Mono), intermediate monocytes (Int. Mono) and non-classical monocytes (NC. Mono) based on CD14 and CD16 expression levels.

C. Monocytes were further characterized based on CD169 expression.

Appendix Table S1. Flow cytometry antibodies per panels

Panel A (100µL of whole blood)

No.	Marker	Colour	Volume (µL)	Clone	Cat. No.	Vendor
1	CD45	BV786	2.5	HI30	304048	BioLegend
2	CD14	PE-CF594	1.5	MOP9	562335	BD Biosciences
3	CD16	APC Cy7	1.5	3G8	302018	BioLegend
4	CD19	Ev605	2.5	SJ25C1	83-0198-42	eBioscience
5	CD11b	BV510	1.5	ICRF44	563098	BD Biosciences
6	CD33	PE-Cy7	1	WM-53	25-0338-42	eBioscience
7	CD169	PE	1.5	7-239	346004	Biolegend
8	HLA-DR	AF700	1.5	L243	307626	Biolegend
9	CD3	APC	1.5	UCHT1	300439	Biolegend
10	CD56	FITC	5	MEM-188	304604	Biolegend
11	CD11c	BV650	2.5	B-ly6	563404	BD Biosciences
12	CD86	BV421	2	2331	562432	BD Horizon
13	CD123	BUV395	2	7G3	564195	BD Horizon
14	CD66b	PerCP cy5.5	2	G10F5	305108	Biolegend

Panel B (100µL of whole blood)

No.	Marker	Colour	Volume (µL)	Clone	Cat No.	Vendor
1	CD3	FITC	1	UCHT1	11-0038-42	eBioscience
2	CD4	BV650	2	SK3	563875	BD Horizon
3	CD8	V500	2	RPA-T8	560774	BD Biosciences
4	CD45RA	PerCP-Cy5.5	2	HI100	304122	Biolegend
5	CD27	APC	2	O323	17-0279-42	eBioscience
6	CD25	PE-Cy7	2	M-A251	557741	BD Biosciences
7	CD127	BUV737	2	HL-7R-M21	564300	BD Biosciences
8	CD38	BUV395	2	HB7	563811	BD Biosciences
9	CD56	PE	2		130-098-756	Miltenyi Biotec
10	CD16	AF700	2	3G8	302036	Biolegend
11	Vd1 TCR	APC-Cy7	1	REA173	130-120-438	Miltenyi Biotec
12	Vd2 TCR	BV711	2	B6	331412	Biolegend
13	VA 7.2 TCR	BV605	2	3C10	351720	Biolegend
14	CD161	BV786	2	HP-3G10	339930	Biolegend
15	CD19	PE-CF594	2	HIB19	562321	BD Biosciences
16	CD57	PB	0.5	HCD57	322316	Biolegend

Panel C (100µL of whole blood)

No.	Marker	Colour	Volume (µL)	Clone	Cat. No.	Vendor
1	CD45RA	PerCP Cy5.5	1	HI100	45-0458-42	eBioscience
2	CD10	FITC	1	HI10a	312208	Biolegend
3	CD11b	PE-Cy7	1	ICRF44	25-0118-42	eBioscience
4	CD49d	PE-CF594	1	9F10	563645	BD Biosciences
5	Siglec8	PE	1	7C9	347104	Biolegend
6	CD8	BV786	0.5	RPA-T8	563823	BD Biosciences
7	CD4	BV650	1	RPA-T4	300536	Biolegend
8	CD16	BV605	1	3G8	563172	BD Horizon
9	CD3	V500	5	UCHT1	561416	BD Biosciences
10	CD66b	BV421	1	G10F5	562940	BD Biosciences
11	HLA-DR	APC-H7	0.5	G46-6	561358	BD Biosciences
12	CCR3	AF647	2	5E8	310710	Biolegend
13	CD38	BUV395	3	HB7	563811	BD Biosciences
14	CD27	BUV737	2	L128	564301	BD Biosciences

Intracellular Panel (1 million PBMCs)

No.	Marker	Colour	Volume (µL)	Clone	Cat. No.	Vendor
1	CD66B	BV421	2	G10F5	562940	BD Biosciences
2	CD45RA	SB436	2	H100	62-0458-42	Thermo Fisher
3	CD27	PB	1	O323	302822	Biolegend
4	NKG2C	BV480	2	134591	748168	BD Biosciences
5	CD8	BV605	1	SK1	564116	BD Biosciences
6	CD19	BV605	1	HIB19	740394	BD Biosciences
7	VD2	BV711	1	B6	331412	Biolegend
8	CD107A	BV785	2	H4A3	563869	BD Biosciences
9	NKP46	BB515	2	9-e2	564536	BD Biosciences
10	CD3	SB550	1	SK7	344852	Biolegend
11	CD169	PERCP5.5	5	7-239	346020	Biolegend
12	HLADR	APCR700	2	L243	307626	Biolegend
13	CD4	SN685	1	SK3	344658	Biolegend
14	CD14	APC CY7	1	M0P9	557831	BD Biosciences
15	VD1	APC Vio770	1	REA173	130-120-578	Miltenyi Biotec
16	L/D	ZOMBIE NIR	0.5		423105	Biolegend
17	CXCR5	PE VIO615	1	J252D4	356928	Biolegend
18	CD154	PE CY5	5	TRAP-1	555701	BD Biosciences
19	CD56	PE CY5.5	2	NCAM16.2	35-0567-42	Thermo Fisher
20	CD16	PEAF700	1	3G8	MHCD1624	Thermo Fisher
21	CD45	BUV805	2	H130	612891	BD Biosciences
22	Granzyme B	BV510	2.5	GB11	563388	BD Biosciences
23	IFN-y	BV570	2.5	4S.B3	502534	Biolegend
24	IL-2	BV650	5	MQ1-17H12	564166	BD Biosciences
25	TNF-a	BV750	2.5	MAB11	566359	BD Biosciences
26	IL-6	FITC	2.5	MQ2-13A5	11-7069-82	Thermo Fisher
27	IL-17a	AF647	10	N49-653	560490	BD Biosciences
28	IL-10	PE	2.5	JES-9D7	501404	Biolegend
29	IL-4	PECY7	2.5	8D4-8	560672	BD Biosciences

Appendix Table S2. Summary of actual P values for Main, Expanded View and Appendix figures.

Figure	Group	P values
**Figure 3B**		
CD4+CD45RA+ Naïve T cells	Asymptomatic vs. Healthy control	0.403
	Asymptomatic vs. Symptomatic	0.084
	Healthy control vs. Symptomatic	0.013
CD4+CD45RA+ TEMRA cells	Asymptomatic vs. Healthy control	0.636
	Asymptomatic vs. Symptomatic	0.511
	Healthy control vs. Symptomatic	0.250
CD4+CD45RA- CM T cells	Asymptomatic vs. Healthy control	0.051
	Asymptomatic vs. Symptomatic	0.942
	Healthy control vs. Symptomatic	0.035
CD4+CD45RA- EM T cells	Asymptomatic vs. Healthy control	0.006
	Asymptomatic vs. Symptomatic	0.046
	Healthy control vs. Symptomatic	0.213
**Figure 3C**		
IL-17a	Asymptomatic vs. Symptomatic	0.008
IFN-γ	Asymptomatic vs. Symptomatic	0.873
TNF-α	Asymptomatic vs. Symptomatic	>0.999
IL-4	Asymptomatic vs. Symptomatic	0.548
IL-10	Asymptomatic vs. Symptomatic	0.151
**Figure 4**		
S-Flow IgG	Asymptomatic vs. Symptomatic	0.558
S-Flow IgM	Asymptomatic vs. Symptomatic	<0.000001
D614 Psuedovirus (IC50)	Asymptomatic vs. Symptomatic	<0.000001
G614 Psuedovirus (IC50)	Asymptomatic vs. Symptomatic	<0.000001
**Figure 5A**		
Mature Neutrophils	Asymptomatic vs. Healthy control	>0.999
	Asymptomatic vs. Symptomatic	0.0121
	Healthy control vs. Symptomatic	0.0041
Immature Neutrophils	Asymptomatic vs. Healthy control	0.000013
	Asymptomatic vs. Symptomatic	>0.999
	Healthy control vs. Symptomatic	<0.000001
**Figure 5B**		
Classical Monocytes	Asymptomatic vs. Healthy control	>0.999
	Asymptomatic vs. Symptomatic	0.002
	Healthy control vs. Symptomatic	0.006
Intermediate Monocytes	Asymptomatic vs. Healthy control	>0.999
	Asymptomatic vs. Symptomatic	0.000089
	Healthy control vs. Symptomatic	0.002
Non-classical Monocytes	Asymptomatic vs. Healthy control	>0.999
	Asymptomatic vs. Symptomatic	>0.999
	Healthy control vs. Symptomatic	>0.999
**Figure 5C**		
CD169+ Classical Monocytes	Asymptomatic vs. Healthy control	>0.999
	Asymptomatic vs. Symptomatic	0.007
	Healthy control vs. Symptomatic	0.003
CD169+ Intermediate Monocytes	Asymptomatic vs. Healthy control	>.999
	Asymptomatic vs. Symptomatic	0.000546
	Healthy control vs. Symptomatic	0.000554
CD169+ Non-classical Monocytes	Asymptomatic vs. Healthy control	0.457
	Asymptomatic vs. Symptomatic	>0.999
	Healthy control vs. Symptomatic	>0.868
**Figure 5E**		
IL-6	Asymptomatic vs. Mild	>0.999
	Asymptomatic vs. Moderate	0/97
	Asymptomatic vs. Severe	0.008

	Mild vs. Moderate	0.98
	Mild vs. Severe	0.005
	Moderate vs. Severe	0.04
IL-7	Asymptomatic vs. Mild	0.07
	Asymptomatic vs. Moderate	0.68
	Asymptomatic vs. Severe	0.95
	Mild vs. Moderate	0.63
	Mild vs. Severe	0.007
	Moderate vs. Severe	0.32
IP-10	Asymptomatic vs. Mild	0.93
	Asymptomatic vs. Moderate	0.19
	Asymptomatic vs. Severe	<0.000001
	Mild vs. Moderate	0.43
	Mild vs. Severe	<0.000001
	Moderate vs. Severe	0.000058
MCP-1	Asymptomatic vs. Mild	>0.999
	Asymptomatic vs. Moderate	0.98
	Asymptomatic vs. Severe	0.02
	Mild vs. Moderate	>0.999
	Mild vs. Severe	0.01
	Moderate vs. Severe	0.06
**Figure 6A**		
BDNF	Asymptomatic vs. Healthy control	<0.00001
	Asymptomatic vs. Symptomatic	0.002
	Healthy control vs. Symptomatic	0.0017
PDGF-BB	Asymptomatic vs. Healthy control	0.0011
	Asymptomatic vs. Symptomatic	<0.000001
	Healthy control vs. Symptomatic	>0.999
VEGF-A	Asymptomatic vs. Healthy control	0.000053
	Asymptomatic vs. Symptomatic	0.000045
	Healthy control vs. Symptomatic	<0.000001
VEGF-D	Asymptomatic vs. Healthy control	<0.000001
	Asymptomatic vs. Symptomatic	<0.000001
	Healthy control vs. Symptomatic	>0.999
VEGF-A/VEGF-D ratio	Asymptomatic vs. Healthy control	<0.000001
	Asymptomatic vs. Symptomatic	<0.000001
	Healthy control vs. Symptomatic	>0.999
**Figure 6C**		
CCR6+ DC	Asymptomatic vs. Healthy control	>0.999
	Asymptomatic vs. Symptomatic	0.0002
	Healthy control vs. Symptomatic	0.0021
CCR7+ DC	Asymptomatic vs. Healthy control	>0.999
	Asymptomatic vs. Symptomatic	0.0009
	Healthy control vs. Symptomatic	0.0399
CXCR5+ DC	Asymptomatic vs. Healthy control	>0.999
	Asymptomatic vs. Symptomatic	0.0343
	Healthy control vs. Symptomatic	0.2038
CXCR5+ CD4 T cell	Asymptomatic vs. Healthy control	>0.9999
	Asymptomatic vs. Symptomatic	0.0071
	Healthy control vs. Symptomatic	0.0647
CXCR5+ CD8 T cell	Asymptomatic vs. Healthy control	0.4442
	Asymptomatic vs. Symptomatic	<0.0001
	Healthy control vs. Symptomatic	0.2389
CXCR5+ B cell	Asymptomatic vs. Healthy control	0.3265
	Asymptomatic vs. Symptomatic	0.1556
	Healthy control vs. Symptomatic	0.0034
CCR7+ B cell	Asymptomatic vs. Healthy control	>0.999
	Asymptomatic vs. Symptomatic	0.0107

	Healthy control vs. Symptomatic	0.0946
**Figure EV1A**		
BDNF	Asymptomatic vs. Mild	0.34
	Asymptomatic vs. Moderate	0.08
	Asymptomatic vs. Severe	0.02
	Mild vs. Moderate	0.80
	Mild vs. Severe	0.57
	Moderate vs. Severe	>0.999
EGF	Asymptomatic vs. Mild	0.338656
	Asymptomatic vs. Moderate	0.079994
	Asymptomatic vs. Severe	0.019839
	Mild vs. Moderate	0.801150
	Mild vs. Severe	0.569166
	Moderate vs. Severe	0.994987
HGF	Asymptomatic vs. Mild	0.993359
	Asymptomatic vs. Moderate	0.984121
	Asymptomatic vs. Severe	<0.000001
	Mild vs. Moderate	0.999376
	Mild vs. Severe	<0.000001
	Moderate vs. Severe	0.000009
VEGF-A	Asymptomatic vs. Mild	0.999998
	Asymptomatic vs. Moderate	0.999001
	Asymptomatic vs. Severe	0.021791
	Mild vs. Moderate	0.998415
	Mild vs. Severe	0.011351
	Moderate vs. Severe	0.043045
VEGF-D	Asymptomatic vs. Mild	0.026805
	Asymptomatic vs. Moderate	0.129801
	Asymptomatic vs. Severe	0.058453
	Mild vs. Moderate	0.974221
	Mild vs. Severe	0.980957
	Moderate vs. Severe	0.999755
**Figure EV1B**		
IL-2	Asymptomatic vs. Mild	0.001188
	Asymptomatic vs. Moderate	0.065225
	Asymptomatic vs. Severe	0.972934
	Mild vs. Moderate	0.728582
	Mild vs. Severe	0.001753
	Moderate vs. Severe	0.110937
IL-5	Asymptomatic vs. Mild	0.318722
	Asymptomatic vs. Moderate	0.002967
	Asymptomatic vs. Severe	0.184265
	Mild vs. Moderate	0.173917
	Mild vs. Severe	0.992123
	Moderate vs. Severe	0.258798
IFN-γ	Asymptomatic vs. Mild	0.999555
	Asymptomatic vs. Moderate	0.980926
	Asymptomatic vs. Severe	0.027277
	Mild vs. Moderate	0.990670
	Mild vs. Severe	0.021920
	Moderate vs. Severe	0.098935
**Figure EV1C**		
IL-1β	Asymptomatic vs. Mild	0.350154
	Asymptomatic vs. Moderate	0.945890
	Asymptomatic vs. Severe	0.832452
	Mild vs. Moderate	0.737023
	Mild vs. Severe	0.035796
	Moderate vs. Severe	0.492324

IL-18	Asymptomatic vs. Mild	0.973723
	Asymptomatic vs. Moderate	0.900403
	Asymptomatic vs. Severe	0.013508
	Mild vs. Moderate	0.989190
	Mild vs. Severe	0.028647
	Moderate vs. Severe	0.124033
**Figure EV2A**		
CD8+ CD45RO- Naive cells	Asymptomatic vs. Healthy control	0.192356
	Asymptomatic vs. Symptomatic	0.315893
	Healthy control vs. Symptomatic	0.002779
CD8+ CD45RO- TEMRA cells	Asymptomatic vs. Healthy control	>0.999
	Asymptomatic vs. Symptomatic	>0.999
	Healthy control vs. Symptomatic	>0.999
CD8+ CD45RO+ CM cells	Asymptomatic vs. Healthy control	>0.999
	Asymptomatic vs. Symptomatic	0.000646
	Healthy control vs. Symptomatic	0.166162
CD8+ CD45RO+ EM cells	Asymptomatic vs. Healthy control	>0.999
	Asymptomatic vs. Symptomatic	0.713786
	Healthy control vs. Symptomatic	>0.999
**Figure EV2C**		
Granzyme+ CD8+ T cells	Asymptomatic vs. Symptomatic	0.841
**Figure EV3**		
IL-17a	Asymptomatic vs. Symptomatic	0.246
IFN-γ	Asymptomatic vs. Symptomatic	0.222
TNF-α	Asymptomatic vs. Symptomatic	0.690
IL-4	Asymptomatic vs. Symptomatic	0.651
IL-10	Asymptomatic vs. Symptomatic	0.690
