# Supplementary material for: Asymptomatic COVID‐19: disease tolerance with efficient anti‐viral immunity against SARS‐CoV‐2
Source: EMBO Mol Med. 2021 May 27;13(6):e14045. doi: 10.15252/emmm.202114045 (PMC8185544; doi:10.15252/emmm.202114045)
Supplement: Supplementary file 2 — Expanded View Figures PDF [file EMMM-13-e14045-s006.pdf]

Expanded View Figures

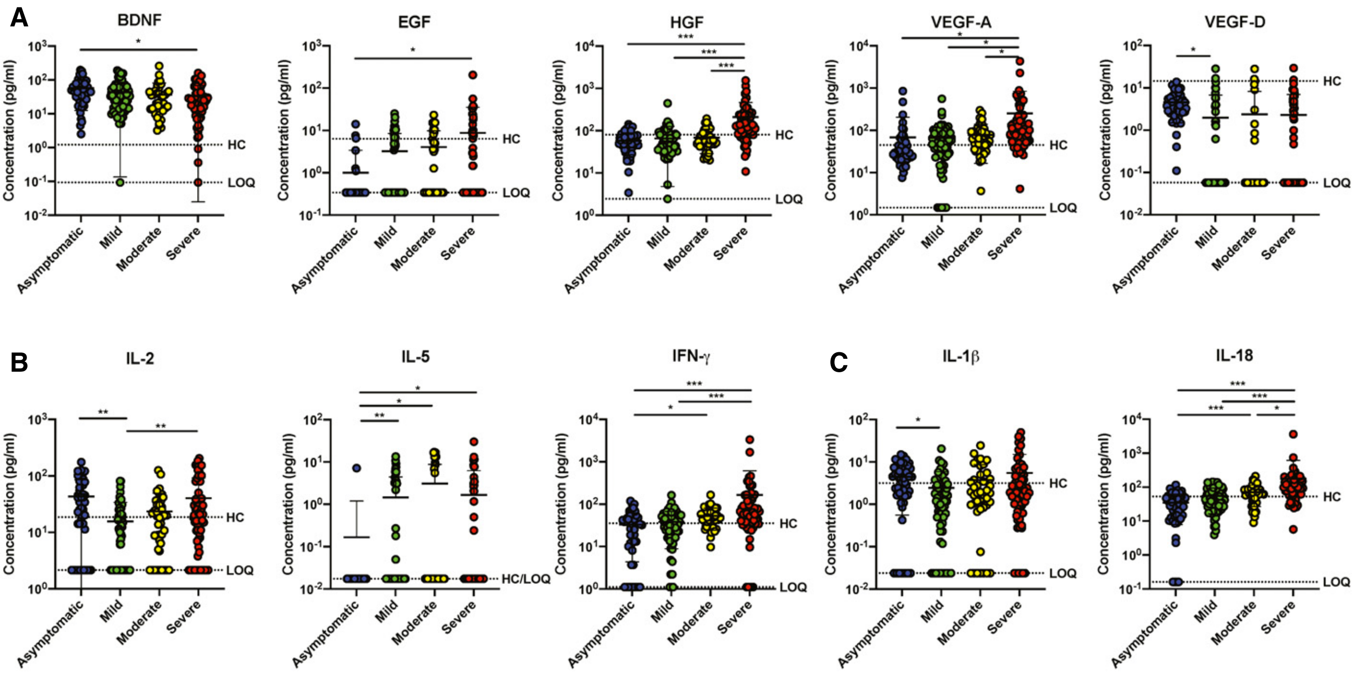

Figure EV1. Signatures of immune mediators in COVID-19 patients of different severity stratum.

A–C (A) Growth factors, (B) T cell-associated immune mediators, and (C) inflammatory macrophage-associated cytokine levels in plasma samples of COVID-19 patients who are asymptomatic (n = 48) or exhibit mild (n = 61), moderate (n = 43), or severe (n = 68) symptoms. Immune mediators were measured in the first plasma sample collected from each SARS-CoV-2 PCR-positive patient. Immune mediator levels for healthy controls (HC) (n = 23) are indicated by the black dotted line. Patient samples with concentrations out of measurement range are presented as the value of limit of quantification (LOQ). Data are presented as mean ± SD. $^{*}P < 0.05$; $^{**}P < 0.01$; and $^{***}P < 0.001$ (one-way ANOVA with post hoc t-test).

Figure EV2. $CD8^{+}$ T-cell responses in COVID-19 patients.

A Mass cytometry was performed on PBMCs obtained from acute symptomatic (n = 37) and acute asymptomatic (n = 19) COVID-19 patients and healthy controls (n = 10). Naïve, TEMRA, central memory (CM), and effector memory (EM) T cells were characterized based on CD45RA and CCR7 expressions. Data are presented as mean ± SD. $^{**}P < 0.01$ and $^{***}P < 0.001$ (Kruskal–Wallis test with Dunn's multiple comparison).

B Representative gating strategy for the characterization of granzyme B expression of $CD8^{+}$ T cells in isolated PBMCs of COVID-19 patients by flow cytometry. Representative gating strategy was performed on a symptomatic patient.

C Comparison of granzyme B expression in $CD8^{+}$ T cells from symptomatic (n = 5) and asymptomatic (n = 5) convalescent PBMCs. Data are presented as mean ± SD (Mann–Whitney U-test).

▶

© 2021 The Authors

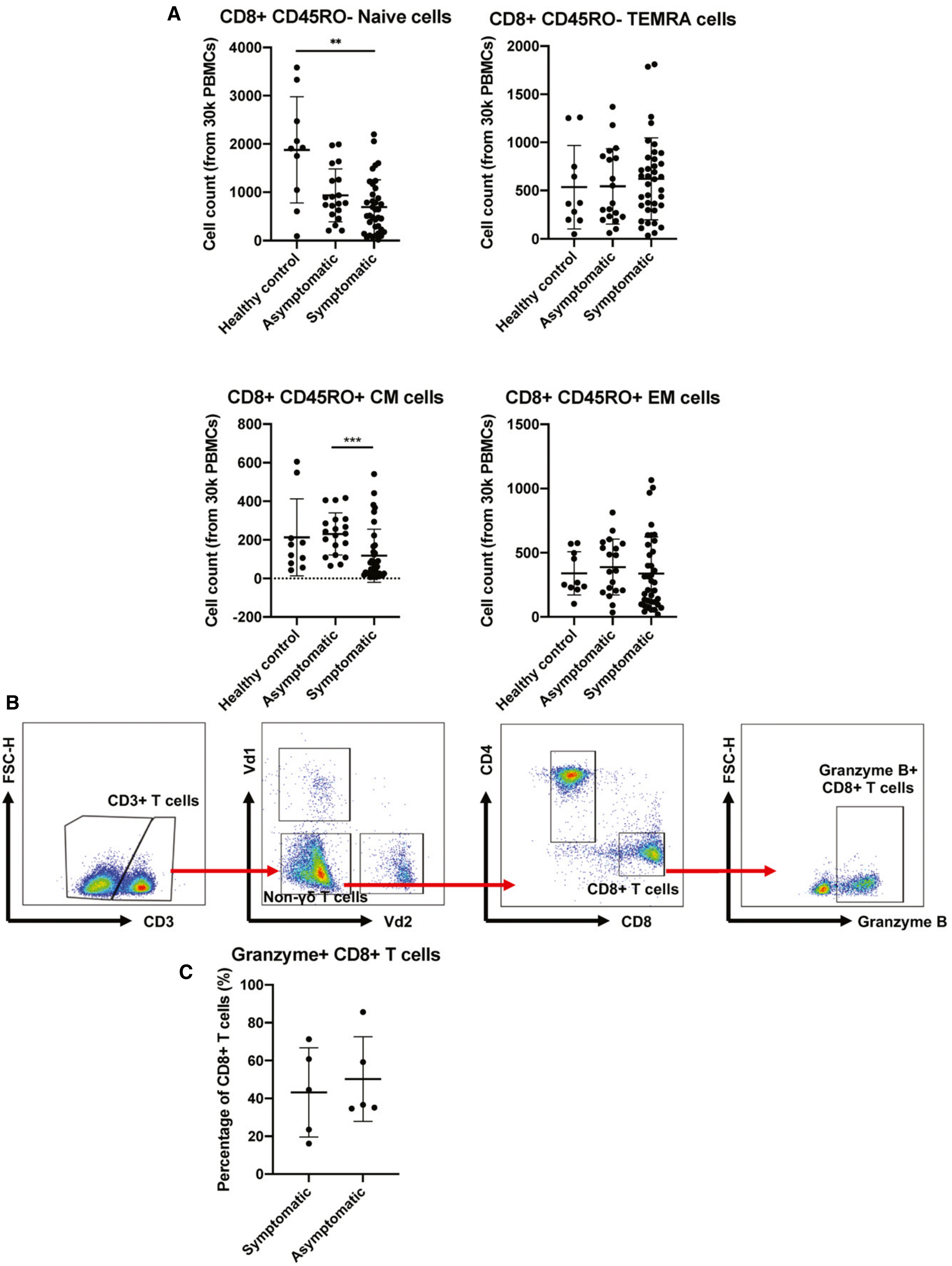
A
CD8+ CD45RO- Naive cells
Cell count (from 30k PBMCs)
**
Healthy control
Asymptomatic
Symptomatic
CD8+ CD45RO- TEMRA cells
CD8+ CD45RO+ CM cells
***
CD8+ CD45RO+ EM cells
B
FSC-H
CD3+ T cells
CD3
Vd1
Non-γδ T cells
Vd2
CD4
CD8+ T cells
CD8
Granzyme B+
CD8+ T cells
Granzyme B
C
Granzyme+ CD8+ T cells
Percentage of CD8+ T cells (%)
Symptomatic
Asymptomatic

Figure EV2.

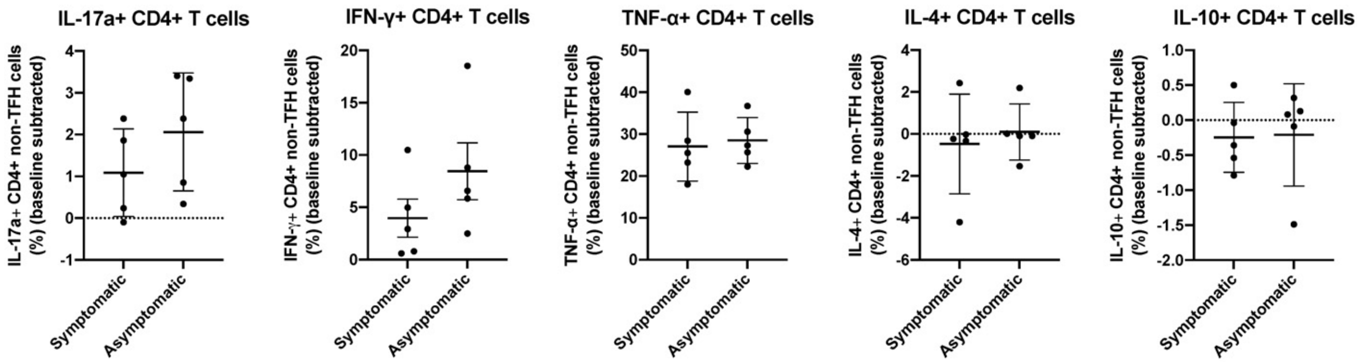

Figure EV3. Asymptomatic patients exhibit similar systemic T-cell responses compared to symptomatic COVID-19 patients.

$CD4^+$ non-T follicular helper (TFH) cells were characterized based on the expression of IL-17a, IFN-γ, TNF-α, IL-4, and IL-10 upon PMA/ionomycin stimulation and compared between symptomatic (n = 5) and asymptomatic (n = 5) patients. Data are presented as mean ± SD (Mann–Whitney U-test).

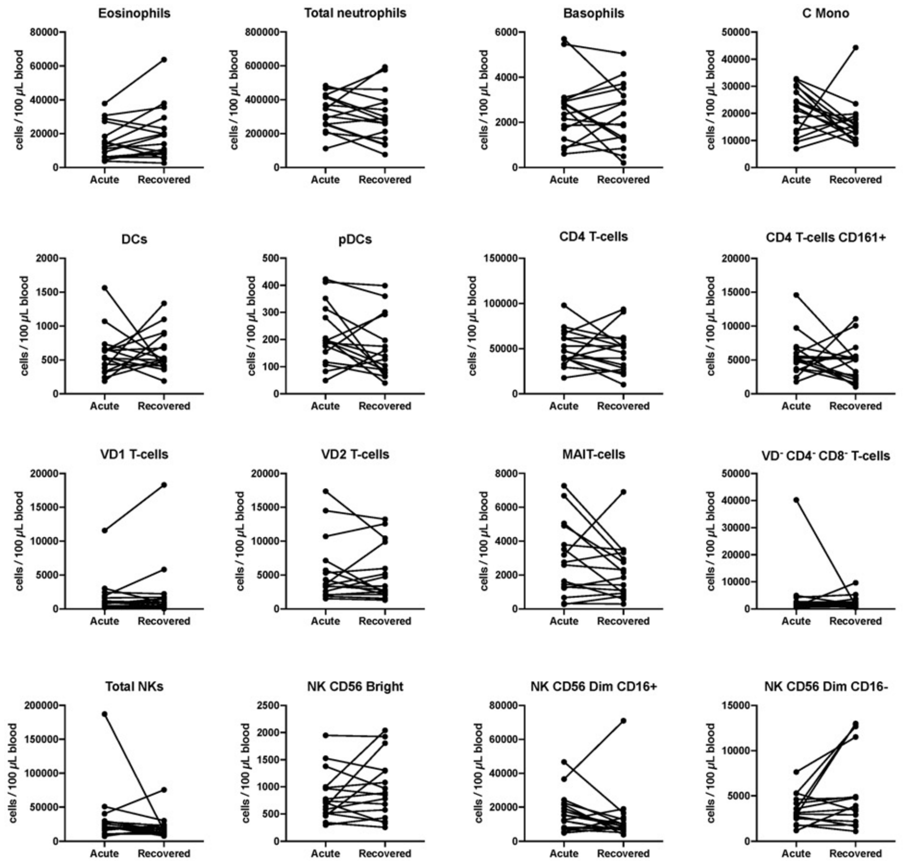

Figure EV4. Circulating immune cells are not impacted during asymptomatic SARS-CoV-2 infection.

Immune cells in 100 μl of blood were quantified by three flow cytometry panels for asymptomatic patients (n = 16) during acute stage and recovered stages. Cells counts between paired samples were compared (non-parametric two-tailed Wilcoxon matched-pairs signed rank test).

© 2021 The Authors
